# Effects of Short Term Exposure of Atrazine on the Liver and Kidney of Normal and Diabetic Rats

**DOI:** 10.1155/2014/536759

**Published:** 2014-09-29

**Authors:** Dinesh Babu Jestadi, Alugoju Phaniendra, Undru Babji, Thupakula Srinu, Bhavatharini Shanmuganathan, Latha Periyasamy

**Affiliations:** Department of Biochemistry and Molecular Biology, School of Life Sciences, Pondicherry University, Puducherry 605014, India

## Abstract

The present study evaluates the effects of short term (15 days) exposure of low dose (300 *μ*g kg^−1^) of atrazine (2-chloro-4-ethylamino-6-isopropylamino-1,3,5-triazine) on antioxidant status and markers of liver and kidney damage in normal (nondiabetic) and diabetic male Wistar rats. Rats were divided into four groups: Group I as normal control, Group II as atrazine treated, Group III as diabetic control, and Group IV as atrazine treated diabetic rats. Atrazine administration resulted in increased MDA concentration as well as increased activities of SOD, CAT, and GPx in both liver and kidney of atrazine treated and atrazine treated diabetic rats. However, GSH level was decreased in both liver and kidney of atrazine treated and atrazine treated diabetic rats. Atrazine administration led to significant increase in liver damage biomarkers such as AST, ALT, and ALP as well as kidney damage biomarkers such as creatinine and urea in both normal and diabetic rats, but this increase was more pronounced in diabetic rats when compared to normal rats. In conclusion, the results of the present study demonstrate that short term exposure of atrazine at a dose of 300 *μ*g kg^−1^ could potentially induce oxidative damage in liver and kidney of both normal and diabetic rats.

## 1. Introduction

Numerous environmental chemicals (ECs) affecting the endocrine activity in humans are included under endocrine disrupting compounds (EDCs) (e.g., polychlorinated biphenyls, bisphenol A, methoxychlor, and atrazine) [1, 2] which are hazardous to the reproductive health of fish and amphibians wild life. But their impact on mammals, particularly humans, is less clear [3]. Atrazine (2-chloro-4-ethylamino-6-isopropylamino-1,3,5-triazine) is a triazine herbicide that is widely used as a preemergence and postemergence herbicide for the control of weeds in maize, sorghum, pineapple, sugar cane, and cereals [4, 5]. Because of its widespread use, residues of atrazine, its metabolites deethylatrazine, and other derivatives contaminated both ground water and surface water for several years. Atrazine is resistant to degradation and has a half-life about 95–350 days [6]. Atrazine exposure is associated with severe health problems such as cancer, neurologic diseases, dermatologic diseases, and respiratory disorders [4]. So the researchers around the globe are mainly concerned and interested to study the adverse effects of atrazine [7].

Diabetes mellitus (DM) is a group of heterogeneous, hormonal, and metabolic disorders characterized by hyperglycemia and glucosuria, resulting from defects in insulin secretion, insulin action, or both [8]. Hyperglycemia can induce the increased production of free radicals both reactive oxygen species (ROS) and reactive nitrogen species (RNS) by various means such as mitochondrial respiratory system, glucose autoxidation and activation of the polyol pathway, formation of advanced glycation end-products (AGEs), and antioxidant enzyme inactivation. At high concentration, free radicals damage major cellular components, including nucleic acids [9], proteins and amino acids [10], and lipids [11]. The prevalence of diabetes varied from 9% to 16.6% in different regions of India, with the southern region having higher prevalence rates than other regions [12]. The aspect of environmental pollution is of great importance, particularly in developing countries, like India, where environmental pollution and diabetes are rampant. Therefore, the present study aimed to investigate the effects of atrazine in normal and diabetic rats.

The primary target of atrazine in humans and animals is the endocrine (hormonal) system. Effects reported in adults (human and experimental animals) include shortening of estrous cycle length, attenuation of the LH (luteinizing hormone) surge, decreases in pituitary hormone levels, ovarian histopathology (changes in ovarian tissue), and liver effects including increased serum lipids and liver enzymes and liver histopathology. Other effects on the central nervous system, immune system, and cardiovascular function have been reported in adults. Exposure to atrazine may be associated with some types of non-Hodgkin's lymphoma in adult humans. Significantly increased risk of preterm delivery, intrauterine growth retardation, and decreased birth weight were associated with atrazine concentrations in drinking water [13].

Pesticides can suppress the expression of functional glucose transporter proteins in several organs, providing a hypothetical mechanism for the observed link between these chemicals and insulin resistance [14]. Exposure to atrazine (ATZ) can cause mitochondrial toxicity and insulin resistance, with even more negative effects observed when animals were fed with high-fat diet [15].

Atrazine (ATZ) is an herbicide which binds irreversibly to the plastoquinone binding sites of photosystem complex II on thylakoid membranes in chloroplasts, thereby inhibiting electron transport. As mitochondrial electron transfer chain (ETC) complexes I and III also have similar Q binding sites, we hypothesized that ATZ might bind to these mitochondrial sites and cause mitochondrial dysfunction. Recently, scientific evidence supports a connection between environmental chemical exposures, which includes herbicides, and development of type 2 diabetes [16]. Prevalence of diabetes among the human population and exposure to toxicants may further aggravate the disease like diabetes. However, there is limited information about the link between influences of atrazine on diabetes. Thus, the purpose of the study was to determine the effects of low concentrations of ATZ on diabetes* in vivo*.

## 2. Materials and Methods

### 2.1. Animals

Adult male Wistar rats, weighing about 120–180 g, were procured from an authorized vendor (Sri Raghavendra Enterprises, Bangalore, India). The animals were housed in plastic cages under standard conditions of 12 h light/12 h dark with an ambient temperature of 24°C ± 2°C. They were fed with normal pelletized chow as diet and water* ad libitum*. The experimental animals handled as per the guidelines of Committee for the Purpose of Control and Supervision on Experiments on Animals (CPCSEA) which were approved by the Institutional Animal Ethics Committee (IAEC approval number: PU/SLS/IAEC/2014/11, dated 20/02/2014) of Pondicherry University, Puducherry, India.

### 2.2. Experimental Plan

Animals were divided into four groups with four rats each. Groups I and II were normal (nondiabetic) rats and Groups III and IV were diabetic rats. Group I (normal control rats-NC): animals were given 300 *μ*L of safflower oil, orally; Group II (atrazine treated rats-NA): animals were given atrazine (300 *μ*g kg^−1^ body weight/rat/day) (Sigma Aldrich, 98.8% purity) dissolved in safflower oil, orally; Group III (diabetic control rats-DC): diabetic animals were given safflower oil, orally; Group IV (atrazine treated diabetic rats-DA): diabetic animals were given atrazine (300 *μ*g kg^−1^ body weight/rat/day) dissolved in safflower oil, orally. The treatment was continued to all the groups for a period of 15 days.

### 2.3. Induction of Diabetes

Diabetes was induced in Groups III and IV rats fed with high fat diet (HFD) for four weeks followed by the intraperitoneal injection of a single dose of streptozotocin (STZ: 35 mg kg body weight). HFD (58% fat, 25% protein, and 17% carbohydrate) was freshly prepared daily in sterile condition with the composition of 365 g normal pelletized rat chow, 310 g lard oil, 250 g casein, 10 g cholesterol, 60 g vitamin, and mineral mix, 3 g methionine, 1 g yeast, and 1 g sodium chloride. After 72 h of STZ induction, animals showing higher blood glucose levels (>140 mg/dL) were considered as diabetic [17, 18].

At the end of the experimental period, the overnight fasted animals were euthanized by cervical dislocation. Blood was collected and allowed to clot, and then the blood samples were centrifuged at 1500 rpm for 15 min to collect the serum. Liver and kidneys were dissected out of rats, washed in ice-cold 1.15% potassium chloride (KCl) solution, and pat-dried and the wet weight was noted. The separated serum and tissues were stored at −80°C until analysis.

### 2.4. Biochemical Parameters

Tissues (liver and kidney) were homogenized in 1x phosphate buffered saline (PBS) pH 7.4 that contained 8 g of sodium chloride (NaCl), 0.2 g of KCl, 1.4 g of disodium hydrogen phosphate (Na_2_HPO_4_), and 0.24 g of potassium dihydrogen phosphate (KH_2_PO_4_), and the homogenate was centrifuged at 10 000 ×g for 15 min at 4°C. The supernatant was collected for analyzing the activities of antioxidant enzymes such as superoxide dismutase (SOD), catalase (CAT), and glutathione peroxidase (Gpx) by S. Marklund and G. Marklund method [19], Claiborne method [20], and Rotruck et al. method [21], respectively. The levels of lipid peroxidation (LPO) and reduced glutathione (GSH) content were analyzed by Ohkawa et al. method [22] and Ellman's [23] method, respectively. Serum parameters like alanine amino transferase (ALT), aspartate amino transferase (AST), alkaline phosphatase (ALP), urea, and creatinine were estimated by commercially available kits (Agappe Diagnostics, Kerala, India). Blood glucose levels were determined by the kit method (Agappe Diagnostics Ltd., Kerala, India). Values were expressed as mg/dL.

### 2.5. Statistical Analysis

Data were analyzed using SPSS software version 16 and one-way analysis of variance (ANOVA) followed by Tukey's test and the results were expressed as mean ± SD; *P* ≤ 0.05 was considered significant.

## 3. Results and Discussion

### 3.1. Effect of Atrazine on Body Weight and Organ Weights

Pesticide exposure usually causes a decrease in body weight and organ weights of animals [23–26]. The changes in the body weight and organ weights of animals are shown in Table 1. In the present study, atrazine administration resulted in increased body weight in all experimental rats during the experimental period. An increase in liver weight and kidney weight was noted in atrazine treated rats compared to normal control rats, whereas increased liver weight but decreased kidney weight was observed in atrazine treated diabetic rats compared to diabetic control rats.

Unchanged or decreased body weights with the administration of atrazine were also reported [27–30]. The decreased body weight and organ weight (i.e., liver and kidney) after atrazine treatment could be due to reduced diet intake or due to necrotic changes in different body tissues [31]. In contrast, in our study, an increased body weight was observed with the atrazine administration. The increase in body weight in our study, particularly in atrazine treated rats (Group II), might be due to increased insulin resistance and hence led to normal weight gain. Our results are similar to a previous study by Gojmerac et al. [32] who have reported increased body weights in rats with the chronic administration of atrazine at low concentrations (30 or 300 *μ*g kg^−1^).

### 3.2. Effect of Atrazine on Lipid Peroxidation

**Figure 1:**
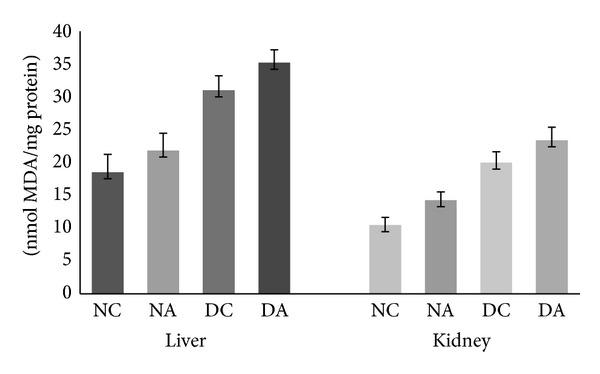
Effect of atrazine on lipid peroxidation. The data are represented as mean ± SD (*n* = 4) and evaluated by one-way analysis of variance (ANOVA) confirmed by Tukey's test. NC normal control rats, NA atrazine treated rats, DC diabetic control rats, and DA atrazine treated diabetic rats.

Atrazine administration resulted in a nonsignificant increase in MDA concentration in both liver and kidney of atrazine treated as well as atrazine treated diabetic rats compared to their respective control groups (Figure 1). Exposure to pesticides is known to induce lipid peroxidation which is responsible for adverse biological effects [14, 33, 34]. Mechanism of pesticide toxicity is usually associated with the increased lipid peroxidation in the liver [35]. Reactive oxygen species such as superoxide anions, hydroxyl radicals, and hydrogen peroxide enhance the oxidative process and induce peroxidative damage to membrane lipids. Though nonsignificant, in the present study an increased LPO was observed in both liver and kidney which might be one of the molecular mechanisms involved in atrazine induced toxicity.

Oxidative stress has been postulated as an important contributing factor in diabetes mellitus [36]. Chronic hyperglycemia induces carbonyl stress which in turn can lead to increased oxidation of lipids [37]. STZ-induced diabetes in rats resulted in increased thiobarbituric acid (TBARS) level [38] which is an indirect evidence of intensified free radical production. The increased concentration of lipid peroxides may propagate oxidative damage by increasing peroxy and hydroxyl radicals.

### 3.3. Effect of Atrazine on Blood Glucose Levels

Significant changes were observed in blood glucose levels in diabetic control and diabetic atrazine rats when compared to normal rats. However, no significant change in the blood glucose levels was observed between diabetic control and diabetic atrazine treated rats (Table 1).

### 3.4. Effect of Atrazine on the Activities of Antioxidant Enzymes (SOD, CAT, and GPx) and on the GSH Content in Liver and Kidney

**Figure 2:**
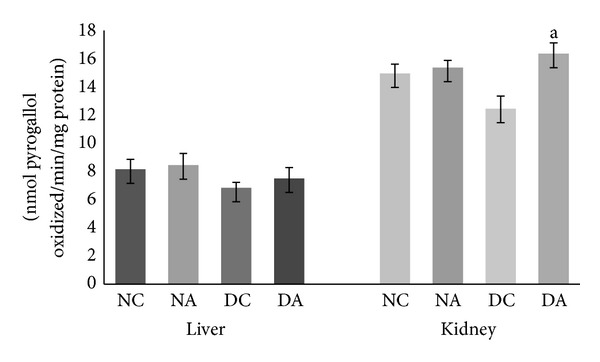
Effect of atrazine on SOD activity in liver and kidney. The data are represented as mean ± SD (*n* = 4) and evaluated by one-way analysis of variance (ANOVA) confirmed by Tukey's test. NC normal control rats, NA atrazine treated rats, DC diabetic control rats, and DA atrazine treated diabetic rats. ^a^ *P* ≤ 0.05 against diabetic control rats.

The antioxidant enzymes including SOD, CAT, and GPx protect against oxidative stress by converting free radicals or reactive oxygen intermediates to nonradical products [39]. SOD provides the first line of defense against oxygen derived free radicals which decreases oxidative stress by dismutation of O^2−^ [40]. It is clearly evident from Figure 2 that atrazine administration led to increased SOD activity in both liver and kidney of atrazine treated as well as atrazine treated diabetic rats compared to normal control and diabetic control rats, respectively. The increased SOD activity was not significant in both liver and kidney of atrazine treated rats compared to normal control rats. However, the increased SOD activity was found to be significant (*P* ≤ 0.05) in the kidney but not significant in the liver of atrazine treated diabetic rats compared to diabetic control rats.

The increase in superoxide dismutase activity after atrazine administration appears to be an adaptive response to increased generation of reactive oxygen species. It has been reported in the literature that exposure of animals to xenobiotics increases SOD activity in various tissues [35].

**Figure 3:**
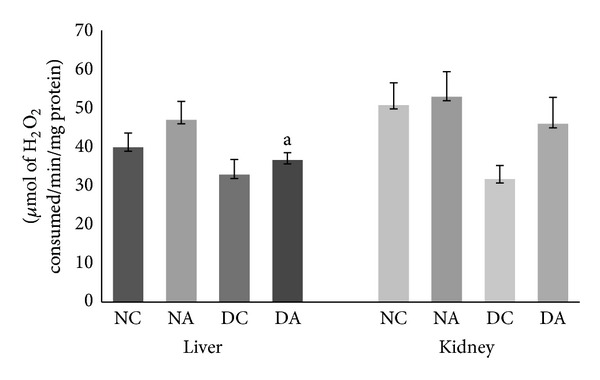
Effect of atrazine on CAT activity in liver and kidney. The data are represented as mean ± SD (*n* = 4) and evaluated by one-way analysis of variance (ANOVA) confirmed by Tukey's test. NC normal control rats, NA atrazine treated rats, DC diabetic control rats, and DA atrazine treated diabetic rats. ^a^ *P* ≤ 0.05 against normal control rats.

**Figure 4:**
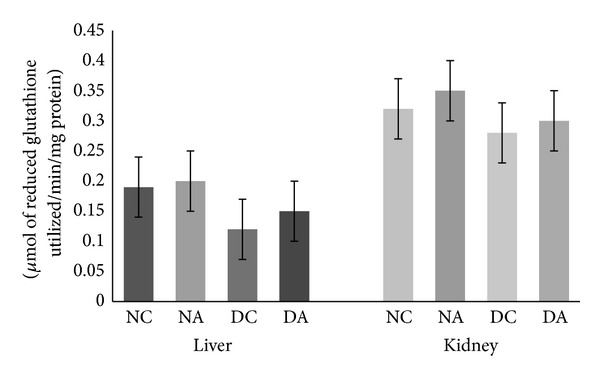
Effect of atrazine on GPx activity in liver and kidney. The data are represented as mean ± SD (*n* = 4) and evaluated by one-way analysis of variance (ANOVA) confirmed by Tukey's test. NC normal control rats, NA atrazine treated rats, DC diabetic control rats, and DA atrazine treated diabetic rats.

Catalase and glutathione peroxidase are the two antioxidant enzymes which remove hydrogen peroxide. Changes in the activities of CAT in liver and kidney are depicted in Figure 3. Atrazine administration also led to increased CAT activity in both liver and kidney of atrazine treated as well as atrazine treated diabetic rats compared to normal control and diabetic control rats, respectively. This increase was significant (*P* ≤ 0.05) only in the liver of atrazine treated diabetic rats compared to diabetic control rats. Changes in the activities of GPx in liver and kidney are depicted in Figure 4. Atrazine administration led to increased GPx activity but not significantly in both liver and kidney of atrazine treated and atrazine treated diabetic rats compared to normal control and diabetic control rats, respectively. The increase in the activities of SOD, CAT, and GPx in this study reflects compensatory mechanism to increased oxidative stress.

**Figure 5:**
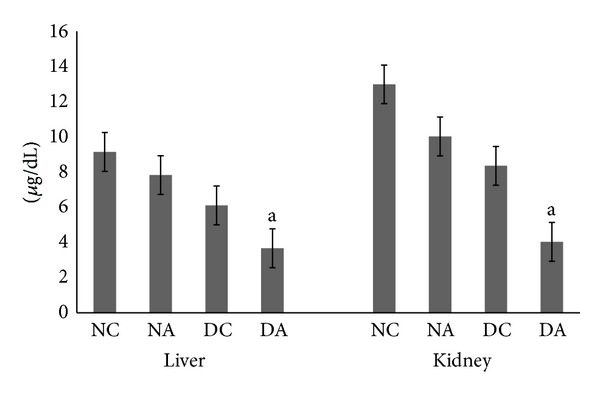
Effect of atrazine on reduced glutathione in liver and kidney. The data are represented as mean ± SD (*n* = 4) and evaluated by one-way analysis of variance (ANOVA) confirmed by Tukey's test. NC normal control rats, NA atrazine treated rats, DC diabetic control rats, and DA atrazine treated diabetic rats. ^a^ *P* ≤ 0.05 against diabetic control rats.

Reduced glutathione (GSH) is the major intracellular thiol that plays a critical role in the cellular defense against oxidative stress. Changes in the level of reduced glutathione content in liver and kidney are depicted in Figure 5. Atrazine administration resulted in a significant (*P* ≤ 0.05) decrease in glutathione content in both liver and kidney of atrazine treated diabetic rats compared to diabetic control rats. Also, decreased GSH content was observed in atrazine treated rats compared to normal control rats, but this decrease was not significant.

Glutathione (GSH) is the most abundant antioxidant in body fluids and tissues. It scavenges the free radicals and thus protects the tissues from oxidative stress. GSH also participates in the detoxification of hydrogen peroxide by various glutathione peroxidases. However, reduced levels of GSH in the liver and kidney were also observed in this study suggesting that administration of low dose of atrazine might contribute to increased oxidative stress and thereby increased tissue damage.

Decrease in GSH levels after administration of various pesticides was observed [41]. A decrease in GSH content in the liver of rats after atrazine exposure indicated prooxidant conditions in the liver.

In conclusion, atrazine induced early hepatic oxidative stress triggered defense mechanisms, that is, increased SOD and CAT activities, to maintain the integrity of the liver.

### 3.5. Effect of Atrazine on Liver and Kidney Damage Biomarkers

Changes in the liver markers (AST, ALT, and ALP) and kidney markers (creatinine and urea) are represented in Table 2. Atrazine administration led to increased levels of liver markers (AST, ALT, and ALP) as well as kidney markers (creatinine and urea) in both atrazine treated and atrazine treated diabetic rats.

The significantly higher AST and ALT activities in animals exposed to atrazine (300 *μ*g kg^−1^ body weight) when compared with control groups are due to the leakage of aminotransferase (AT) enzymes from injured liver cells. These results are similar to other studies [42]. On the contrary, another study reported that rats treated with 400 mg kg^−1^ atrazine for 14 consecutive days resulted in nonsignificant elevation in serum ALT enzyme [43]. ALT is thought to be more specific for hepatic injury because it is present mainly in the cytosol of the liver and in low concentrations elsewhere [44]. The elevation of ALT in the current study was attributed specifically to the injury of liver cells caused by atrazine [42], whereas the AST is a mitochondrial enzyme found in the heart, liver, skeletal muscle, and kidney and is normally present in plasma [45]. The elevated serum AST is apparently due to mitochondrial damage by reactive oxygen species (ROS) induced by atrazine [46].

Atrazine exposure resulted in a significant increase in serum creatinine and urea in atrazine treated and atrazine treated diabetic rats when compared to normal control rats and diabetic control rats, respectively. Nephrotoxicity of atrazine is a consequence of its elimination through the kidneys which leads to a decrease in creatinine clearance and proteinuria. These results are in agreement with the previous report by Liu et al. [47].

An increase in the activity of ALP in serum may reflect pathologic changes in the liver. The serum level of both ALP and ALT is used as a marker of the cell membrane integrity. The injurious effects of atrazine may result from its generation of ROS that causes oxidative stress of various organs. Increased oxidative stress and lipid peroxidation are implicated in the pathogenesis of herbicide-induced hepatic injury [48].

## 4. Conclusion

From the above results, it can be concluded that exposure to atrazine at low dose (300 *μ*g kg^−1^) for a short time period can induce oxidative stress and damage the liver and kidney of both normal and diabetic rats

## Figures and Tables

**Table 1:** Effect of atrazine on the body weight and weights of the organs (liver and kidney).

Weight (in grams)	NC	NA	DC	DA
Initial body weight	163.33 ± 5.77	170.66 ± 5.13	140.00 ± 8.75	152.25 ± 5.90
Final body weight	242.33 ± 4.93	272.66 ± 14.18	193.25 ± 11.44	217.75 ± 6.75
Liver weight	7.81 ± 0.54	10.28 ± 0.68	8.29 ± 0.69	9.07 ± 0.78
Kidney weight	1.61 ± 0.16	1.79 ± 0.46	1.93 ± 0.28	1.79 ± 0.13
Blood glucose∗	89.73 ± 1.05	90.89 ± 3.04	255.33 ± 9.60	268.67 ± 2.89

The data are represented as mean ± SD (*n* = 4) and evaluated by one-way analysis of variance (ANOVA) confirmed by Tukey's test. NC normal control rats, NA atrazine treated rats, DC diabetic control rats, and DA atrazine treated diabetic rats. ∗Blood glucose level values are in mg/dL. Significant difference (*P* < 0.05) is observed only in blood glucose levels of normal control rats and normal atrazine treated rats compared with diabetic control rats and diabetic atrazine treated rats.

**Table 2:** Effect of atrazine on the liver and kidney marker enzymes.

Source	Parameters	NC	NA	DC	DA
Liver markers (IU/L)	AST	24.90 ± 2.26	35.75 ± 2.47∗	49.73 ± 3.60	60.64 ± 3.42∗∗
	ALT	14.85 ± 1.20	20.56 ± 2.34∗	33.02 ± 2.29	45.33 ± 2.53∗∗
	ALP	38.38 ± 2.78	50.68 ± 3.6∗	61.60 ± 4.6	71.41 ± 3.32∗∗

Kidney markers (mg/dL)	Creatinine	0.4 ± 0.07	1.05 ± 0.21∗	1.95 ± 0.07	2.12 ± 0.17∗∗
	Urea	33.05 ± 1.58	42.21 ± 1.70∗	56.21 ± 1.40	59.45 ± 1.86∗∗

The data are represented as mean ± SD (*n* = 4) and evaluated by one-way analysis of variance (ANOVA) confirmed by Tukey's test. NC normal control rats, NA atrazine treated rats, DC diabetic control rats, and DA atrazine treated diabetic rats.

*P ≤ 0.05 (normal control rats versus normal atrazine treated rats).

**P ≤ 0.01 (diabetic control rats versus atrazine treated diabetic rats).
